# A comparative analysis of lung function and spirometry parameters in genotype-controlled natives living at low and high altitude

**DOI:** 10.1186/s12890-022-01889-0

**Published:** 2022-03-21

**Authors:** Esteban Ortiz-Prado, Sebastián Encalada, Johanna Mosquera, Katherine Simbaña-Rivera, Lenin Gomez-Barreno, Diego Duta, Israel Ochoa, Juan S. Izquierdo-Condoy, Eduardo Vasconez, German Burgos, Manuel Calvopiña, Ginés Viscor

**Affiliations:** 1 One Health Research Group, Faculty of Medicine, Universidad de las Américas, Calle de los Colimes y Avenida De los Granados, 170137 Quito, Ecuador; 2 Department of Cell Biology, Physiology and Immunology, Universidad de Barcelona, Barcelona, Spain; 3 Limoncocha Community Health Unit, Limoncocha, Ecuador; 4 Oyacachi Community Health Unit, Oyacachi, Ecuador; 5 Faculty of Medicine, Universidad de las Américas, Quito, Ecuador

**Keywords:** Limoncocha, Oyacachi, Kichwas, Spirometry, Pulmonary function, High altitude

## Abstract

**Background:**

The reference values for lung function are associated to anatomical and lung morphology parameters, but anthropometry it is not the only influencing factor: altitude and genetics are two important agents affecting respiratory physiology. Altitude and its influence on respiratory function has been studied independently of genetics, considering early and long-term acclimatization.

**Objective:**

The objective of this study is to evaluate lung function through a spirometry study in autochthonous Kichwas permanently living at low and high-altitude.

**Methodology:**

A cross-sectional study of spirometry differences between genetically matched lowland Kichwas from Limoncocha (230 m) at Amazonian basin and high-altitude Kichwas from Oyacachi (3180 m) in Andean highlands. The sample size estimates permitted to recruited 118 patients (40 men and 78 women) from Limoncocha and 95 (39 men and 56 women) from Oyacachi. Chi-square method was used to analyze association or independence of categorical variables, while Student’s t test was applied to comparison of means within quantitative variables. ANOVA, or in the case that the variables didn’t meet the criteria of normality, Kruskal Wallis test were used to compare more than two groups.

**Results:**

The FVC and the FEV_1_ were significantly greater among highlanders than lowlanders (*p* value < 0.001), with a proportion difference of 15.2% for men and 8.5% for women. The FEV_1_/FVC was significantly higher among lowlanders than highlanders for men and women. A restrictive pattern was found in 12.9% of the participants.

**Conclusion:**

Residents of Oyacachi had greater FVC and FEV_1_ than their peers from Limoncocha, a finding physiologically plausible according to published literature. Lung size and greater ventilatory capacities could be an adaptive mechanism developed by the highlander in response to hypoxia. Our results support the fact that this difference in FVC and FEV_1_ is a compensatory mechanism towards lower barometric and alveolar partial pressure of oxygen pressure.

## Introduction

Worldwide, more than 140 million people reside above 2500 m [1]. Studying high-altitude dwellers is essential to understand the environmental, physiological and genetic factors that are linked to the incidence and prevalence of different maladies in these populations [2]. Acute and chronic exposure to high altitude has a variety of effects on human physiology and diseases occurrence [3]. Barometric pressure decreases exponentially with increasing altitude [4]. Consequently, the partial pressure of oxygen also decreases, despite the composition of gases in the atmosphere remains unaltered. The physiological consequences of this reduction in oxygen availability begin to be noticeable, even at rest, from an altitude of 2500 m [4].

Living at high altitudes requires different genetical, molecular, physiological and anatomical adaptive mechanisms to counteract the effects of acute or chronic hypoxia [5–7]. Those mechanisms or changes that attempt to improve the ventilatory and cardiovascular responses are likely to be the most significant [8, 9]. Anatomical changes including chest depth and chest width have been described among high altitude natives [10–13]. Improved hypoxic ventilatory responses (HVR) and differences in ventilations rates have also been described among high altitude population [14–16]. All these changes in general translate into a better ventilation and improved spirometric values when compared with individuals living at sea level [17, 18]. Among healthy people living at different altitudes, it has been shown that lung pulmonary volumes is generally higher in individuals living at high altitudes than in those living at sea level [19]. Pulmonary function seems to relate to the time that elapses between exposure to hypoxia and the performance of the spirometry. For instance, in non-adapted subjects who are acutely exposed to high altitudes, lung capacity decreases temporarily. Compte-Torrero et al., reported that expiratory volume during the first second of forced expiration (FEV_1_) was reduced by 12.3% (± 5.7%) and the mean Forced vital capacity (FVC) by 7.6% (± 6.7%) among 8 mountaineers who ascended to 3000 m [20]. They also found that the FEV1/FVC ratio, also called Tiffeneau–Pinelli index remained normal [20]. Another short term exposure analysis performed by Cremona et al. at 1200 m and 4559 m of showed that the FVC did not present significant variations but the FEV_1_ increased by 2% among the high altitude group [21].

In terms of long-term exposure, several studies show people who live at high altitudes have significantly higher spirometric values than those who live at sea level [18]. According to Weitz et al., people who reside above 3200 m have greater FVC and FEV_1_ parameters than those low altitude dwellers [22]. Similarly, Brutsaert et al., compared the results of two Bolivian populations located at 3600 m and 420 m of elevation [11]. They found greater FVC and FEV_1_ volumes when comparted to the subjects located at a lower elevations [11].

In this sense, our study aimed to compare the predicted spirometric values derived from the two genotyped-controlled indigenous populations. One located at 230 m of elevation (Limoncocha) and the other one located at 3800 (Oyacachi).

## Methodology

### Study design

A cross-sectional analysis of the differences in spirometric parameters was carried out in two populations of Kichwa natives from Ecuador living at two different elevations.

### Setting

This study was carried out in Ecuador in two geographically different areas, the Andes and the Amazon Basin. The research work began in January 2017 and concluded in August 2019.

**Fig. 1:**
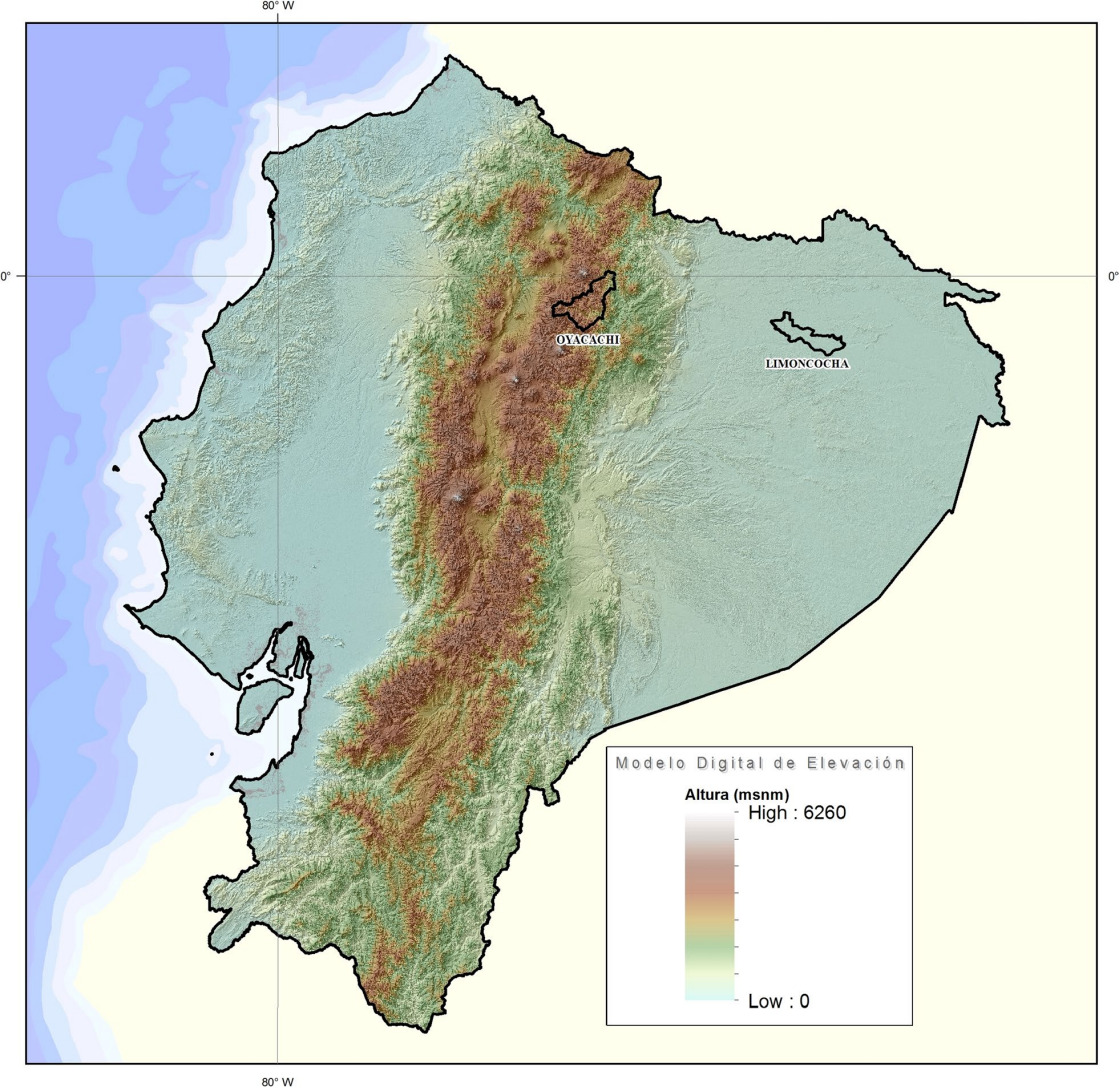
Topographic map of Ecuador highlighting Limoncocha (230 m) and Oyacachi (3800 m)

Ecuador with an area of more than 283,000 km^2^ is the smallest country in the Andean mountainous region in South America. The country is divided into 4 geographical regions, the coast, the highlands, the Amazon region, and the Galapagos Islands. The political division encloses 24 provinces, 10 from the highlands, 7 from the coast, 6 from the Amazon region and 1 from the insular region of Galapagos. Every province has several political divisions called cantons and they are comparable to cities elsewhere. The country has 141 cantons at low altitude, 28 at moderate altitude, 41 at high altitude and 11 at very high altitude. Limoncocha is located at low altitude while Oyacachi is located at very high altitude (Fig. 1).

### Participants

The study was carried out with the voluntary participation of 71 members of the Oyacachi community (high-altitude group) located at 3800 m of elevation and 76 people from Limoncocha (low-altitude control group) located at 230 m of altitude. All the participants who voluntarily agreed are members of the Kiwcha indigenous group in Ecuador.

### Inclusion criteria

Healthy volunteers of both sexes between the ages of 21 and 75 who were born and currently reside in Oyacachi (high-altitude group) and Limoncocha (low-altitude control group).

### Exclusion criteria

Volunteers under 21 years of age, born in another community and who do not normally reside in the parishes were excluded from the study, in addition to those with a history of acute coronary syndrome, acute retinal detachment, aortic aneurysm, endocranial hypertension, and pneumothorax less than 1 month.

### Variables

Sociodemographic variables such as age, sex, place of residence was recorded. General anthropometric measurements including weight, height and BMI were obtained. The following spirometry parameters: FEV_1_, FVC, FEF25–75% and PEF were included according to predicted values for reference population, meanwhile FEV_1_/FVC, known as Tiffeneau-Pinelli index was calculated according to observed values [23].

### Predicted values

To avoid volumetric differences among a variety of subjects, spirometric predictive values were calculated in relation to the anthropometric measurements of each patient (weight, height, and age).

Each spirometry was interpreted according to values considered as normal, based on the comparison of the values obtained by a patient with those that would theoretically correspond to a healthy individual with the same anthropometric characteristics. This theoretical value or reference value (RV) is estimated based on prediction equations (PE) for a Latin American population.

### DNA extraction and analysis of ancestry ratios

To compare the ancestry of the two populations, a subsample of 47 unrelated individuals (30 Oyacachi vs. 17 Limoncocha) was selected. The methodology is amply explained by Ortiz-Prado et al., 2021 [7].

### Exposure

The chronic exposure to high altitude among indigenous people living in Oyacachi located at 3800 m.

### Outcome

To compare the predicted spirometric values between exposed (high altitude) and not exposed (low altitude) participants.

### Data sources

Individual-level socio-demographic information, place of residence and past medical history was obtained in-situ in both communities. A physical examination including body weight, height and weight was performed. Pulmonary function variables were obtained by performing spirometry in all participants FEV_1_, FVC, FEV1/FVC, FEF25–75% and PEF were recorded.

### Study size and sample size calculation

In terms of the number of patients required to achieve significance the sample size (*n*) and margin of error (*E*) were given by the following formula:

$$\begin{aligned} x & = Z\left( {c/100} \right)^{2} r\left( {100 - r} \right) \\ n & = N \, x/((N - 1)E^{2} + x) \\ E & = {\text{Sqrt}}\left[ {\left( {N - n} \right)x/n(N - {1})} \right] \\ \end{aligned}$$

where *N* is the population size (n = 570 in Oyacachi and n = 890 in Limoncocha), (*r*) is the fraction of predicted responses (50%), and *Z*(*c*/100) is the critical value for the confidence level (*c*)*.* The total number of medical and physical evaluation required to achieve significance was 82 for the high altitude group and 96 for the low-altitude control group. The total number of medical and physical evaluations required to achieve statistical significance was 82 for the high-altitude group and 96 for the low-altitude group. Through a non-probability convenience-based sampling technique 118 patients (40 men and 78 women) were included in Limoncocha and 95 (39 men and 56 women) for Oyacachi. A sub-group analysis of only those who met the inclusion criteria for spirometry were included within our study.

### Data analysis

Descriptive statistics were used to analyze and visualize differences between the two populations. T-tests were used to analyze differences between continue variables and Chi square test were the association or independence of categorical variables. When the predicted values were less than 5 in any of the categories, Fisher's exact test or Spearman's test were used when the variable had evident asymmetries with histograms prior to the selection of the test. The strength of association between categorical variables was performed using the V-Cramer test.

All statistical analysis accepted significance with a *p* value < 0.05. Calculations were completed using the IBM Corp. Released 2014. IBM SPSS Statistics for Windows, Version 24.0. Armonk, NY: and R Core Team software 2018 version 3.5.1. Cartography was generated using QGIS Development Team 2.8 and all the references were managed using the open source software Zotero 5.0.85.

### DNA extraction and analysis of ancestry ratios

To compare the ancestry of the two populations, a subsample of 47 unrelated individuals (30 Oyacachi vs. 17 Limoncocha) was selected. We looked for a subsample among all the individuals to identify those subjects who did not have any first order degree of consanguinity, condition that is based on our laboratory protocol for ancestry analysis. DNA extraction was performed from FTA cards (GE Healthcare) by the Chelex method, then the extracts were diluted to a concentration of 5 ng/ul using the NanoDrop 2000 UV–Vis spectrophotometer (Thermo Scientific, Waltham, MA) [24]. 46-plex autosomal ancestry informative deletion-insertion markers (46-plex AIMs-InDel) were amplified. Fluorescent amplicons were sized by capillary electrophoresis in Pop-7 polymer using a genetic analyzer ABI 3130 (Applied Biosystems, Austin, TX). Alleles were named by the software Genemapper V 3.1 (Life Technologies, Carlsbad, CA) following nomenclature described by Pereira et al., 2012 [25]. Taking into account tri-hybrid historic mixture in Ecuador [26–28], Inference of ancestry proportions were obtained considering the admixture model with K = 3 (based in Runs consisted of 100,000 burnin steps, followed by 100,000 Markov Chain Monte Carlo (MCMC) using STRUCTURE V2.3.4 software [29].

All runs were made without any prior information on the origin of samples and only considered the genetic background for the ancestral continental populations based on reference samples: European, EUR (n = 158); African, AFR (n = 105); and Native American, NAM (n = 64). Reference genotypes were extracted from the diversity panel of the Human Genome Diversity Project-Center d'Etude du Polymorphisme Humain (HGDP-CEPH). The populations selected as comparative groups for Africa were: Angola (n = 1), Botswana (n = 4), Central African Republic (n = 23), Congo (n = 13), Kenya (n = 11), Lesotho (n = 1), Namibia (n = 6), Nigeria (n = 22), Senegal (n = 22) and, South Africa (n = 2); for South America: Brazil (n = 22), Colombia (n = 7), and Mexico (n = 35); and for Europe were: France (n = 52), Italy (n = 49), Orkney Islands (n = 15) and Russia (n = 42).

## Results

We included 213 patients from both communities, 69% (n = 118) were from Limoncocha and 31% (n = 95) from Oyacachi. The spirometry subgroup included 147 patients, 52% (n = 76) from the low altitude group and 48% (n = 71) from the high altitude group.

### Age and sex differences

Within our cohort, women were one year younger (36.5 y/o) than men (37.5 y/o), while within sex, those living at low altitude were on average of 38 y/o, and those from the high altitude group were 35 y/o, none of these differences were statistically significant (Table 1).

**Table 1:** Sociodemographic, anthropometric and risk factors analysis from the low and high cohorts

	Female				Male			
	Low altitude	High altitude	(%) Diff	Sig.	Low altitude	High altitude	(%) Diff	Sig.
Age (mean)	38.0 (28.0–48.0)	35.0 (30.0–41.0)	3.00	0.596	41.0 (29.0–50.0)	34.0 (27.0–52.0)	4.33	0.319
Age categories								
Young adult	28 (71.8)	33 (80.5)	16.40	0.641	23 (62.2)	22 (73.3)	4.44	0.348
Adult	10 (25.6)	7 (17.1)	35.29	0.641	12 (32.4)	8 (26.7)	40.00	0.348
Elderly	1 (2.6)	1 (2.4)	0.00	0.641	2 (5.4)	0 (0.0)		0.348
Weight (kg)—median (IQR)	64.0 (57.0–75.0)	59.0 (55.0–66.0)	5.00	**0.035**	73.0 (66.0–80.0)	64.5 (60.0–67.5)	8.50	**0.001**
Height (cm) (m ± SD)	1.51 (0.05)	1.51 (0.06)	0.00	0.676	1.61 (0.06)	1.61 (0.05)	0.00	0.859
BMI—median (IQR)	28.2 (25.0–31.4)	26.5 (24.0–27.6)	1.70	**0.015**	27.6 (25.8–30.0)	24.8 (23.2–26.0)	2.80	**0.041**
BMI categories								
Under weight	0 (0.0)	0 (0.0)		**0.048**	0 (0.0)	0 (0.0)		**0**
Normal	9 (23.1)	13 (31.7)	36.36	**0.048**	4 (10.8)	16 (57.1)	120.00	**0.001**
Over weight	17 (43.6)	23 (56.1)	30.00	**0.048**	23 (62.2)	11 (39.3)	70.59	**0.001**
Obesity	7 (17.9)	5 (12.2)	33.33	**0.048**	7 (18.9)	1 (3.6)	150.00	**0.001**
Extreme obesity	6 (15.4)	0 (0.0)		**0.048**	3 (8.1)	0 (0.0)		**0.001**
Total	39 (100.0)	41 (100.0)	5.00	**0.048**	37 (100.0)	28 (100.0)	27.69	**0.034**
Smoking								
Yes	0 (0.0)	2 (4.9)		0.162	0 (0.0)	4 (13.3)		**0.036**
No	39 (100.0)	39 (95.1)	0.00	0.162	37 (100.0)	26 (86.7)	34.92	**0.036**
Total	39 (100.0)	41 (100.0)	5.00	0.162	37 (100.0)	30 (100.0)	20.90	**0.036**
Lung disease								
Yes	0 (0.0)	3 (7.3)		0.085	0 (0.0)	4 (13.3)		**0.036**
No	39 (100.0)	38 (92.7)	2.60	0.085	37 (100.0)	26 (86.7)	34.92	**0.036**
Total	39 (100.0)	41 (100.0)	5.00	0.085	37 (100.0)	30 (100.0)	20.90	0.036

Bold indicates a *p*-value less than < 0.05 is statistically significant

*IQR* interquartile range, *(m* ± *SD)* mean and standard deviation, *BMI* body mass index

Men living at low altitude where on average 7 years older than men living at high altitude nevertheless, this difference was not significant.

### Weight, height, and BMI

In relation to weight, women from the low altitude location were on average 5% heavier than those women living at high altitude (*p*: 0.035), while low altitude men were 8.5% heavier than their high altitude counterpart (*p* < 0.001) In terms of height, both men and women had an average height of 151 cm and 161 cm respectively, having no differences within groups (Table 1).

### Measured spirometric results

The high altitude group have a greater FVC and the FEV_1_, nevertheless, these differences are not significant. The FEV_1_/FVC ratio was inferior at the high altitude group as well as the forced expiratory flow measurements (Table 2).

**Table 2:** Measured spirometric values between low and high altitude locations

Variables	Female			Male		
	Low altitude	High altitude	(%) Diff	Low altitude	High altitude	(%) Diff
FVC (L)	2.62 (0.41)	2.94 (0.41)	11.50%	3.54 (0.69)	3.96 (0.64)	11.20%
FEV1 (L)	2.55 (0.49)	2.73 (0.43)	6.81%	3.35 (0.65)	3.61 (0.54)	7.47%
FEV_1_/FVC %	95.44 (4.48)	92.76 (5.87)	-2.84%	94.93(5.24)	91.35 (4.78)	−3.84%
FEF 25-75% (L/s)	3.90 (0.95)	3.95 (0.91)	1.27%	4.74 (1.37)	4.55 (0.89)	−4.09%
PEFR (L/s)	5.67(1.06)	5.87 (1.27)	3.46%	7.83 (1.44)	7.81 (1.38)	0.25%

FVC (forced vital capacity), forced expiratory volume in 1 s (FEV1) ml, forced vital capacity (L), forced expiratory volume in 1 s (FEV1) (L), the FEV1/FVC ratio, also called Tiffeneau–Pinelli index) %, forced expiratory flow at 25–75% of forced vital capacity (FVC) (FEF25–75%) (L/s), forced expiratory flow at 25–75% of forced vital capacity (FVC) (FEF25–75%—85%) L/s, peak expiratory flow (L/s), body mass index (BMI) (kg/m^2^)

### Predicted spirometric results

The predicted values demonstrate that high altitude dwellers have greater FVC for both men and women. Also, when compared to the predicted values, the FEV1 was greater for both sexes although only significant among women (Table 3).

**Table 3:** Predicted spirometry values according to sex and weight by altitude

Variables	Female				Male			
	Low altitude	High altitude	(%) Diff	Sig.	Low altitude	High altitude	(%) Diff	Sig.
FVC (%)	93.51 (15.40)	108.93 (19.70)	15.23%	**0.001**	92.24 (15.16)	100.43 (15.56)	8.50%	**0.033**
FEV1 (%)	101.36 (23.03)	115.15 (14.92)	12.74%	**0.002**	104.32 (16.48)	105.00 (24.02)	0.65%	0.892
FEV1/FVC (Ratio)	1.18 (1.11–1.23)	1.14 (1.10–1.18)	−4.0%	**0.029**	1.20 (1.15–1.24)	1.14 (1.10–1.18)	−6.0%	**0.003**
FEF 25-75% (%)	110.32 (26.75)	112.54 (24.88)	1.99%	0.703	114.25 (23.45)	109.07 (21.65)	−4.64%	0.358
PEFR (%)	94.00 (17.46)	97.17 (18.32)	3.31%	0.431	95.46 (16.60)	96.03 (26.26)	0.60%	0.914

Bold indicates a *p*-value less than < 0.05 is statistically significant

*IQR* interquartile range, *(m* ± *SD)* mean and standard deviation

**Fig. 2:**
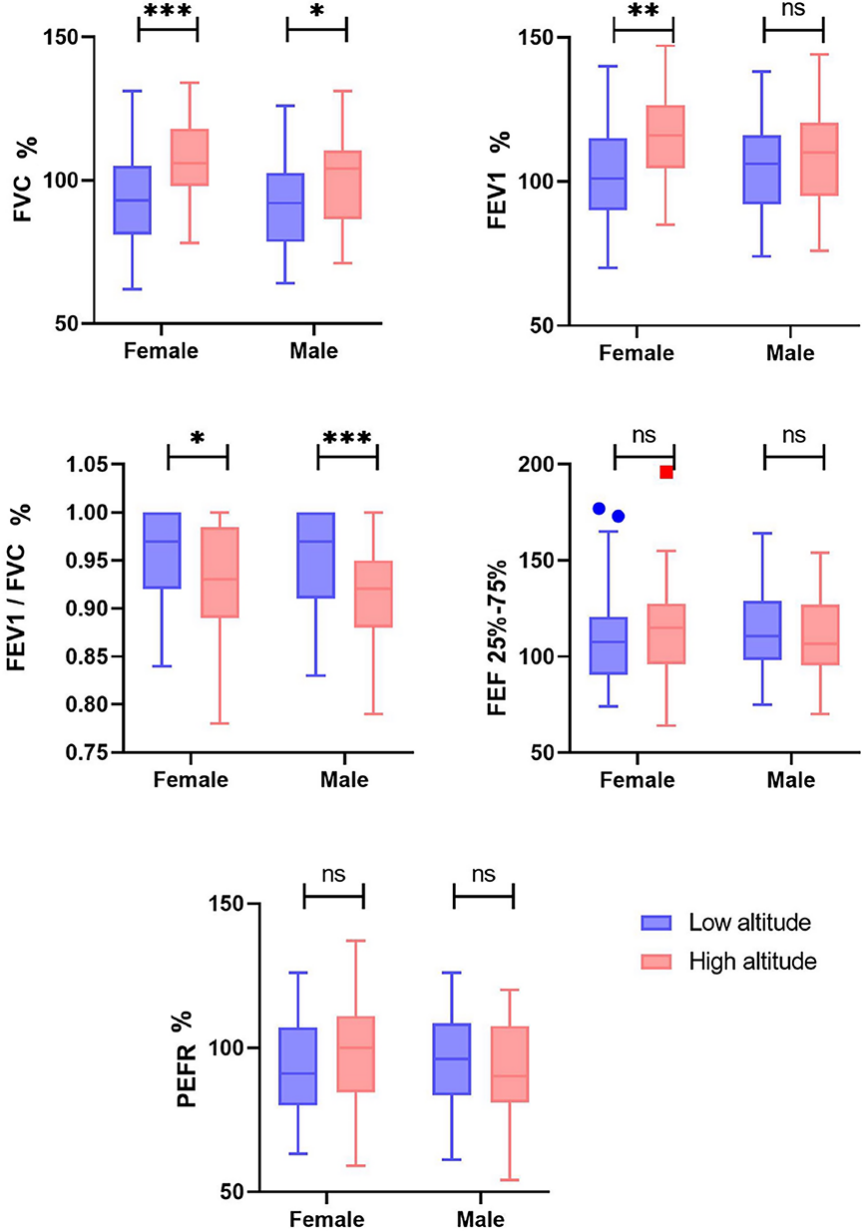
Spirometric values compared to predicted values among low and high altitude dwellers

The maximum flow rate generated during a forceful exhalation (PEFR) was also greater among highlanders when compared to the predicted values, nevertheless, the differences where not significant (Fig. 2).

## Discussion

In our study we found that high altitude dwellers have greater FVC and the FEV_1_, similar to those results published by other authors [11, 17–19]. We found that people living at high altitudes have a higher forced vital capacity than those living at low altitudes, and this difference is statistically significant for both men and women (Fig. 2).

One of the most studied environmental factors related to lung function is altitude exposure and the subsequent hypoxia [4]. Although several studies have discussed this, the role that low barometric pressure (BP) may have directly on lung expandability has rarely been analyzed [30]. Visiting or residing at high altitudes is accompanied by a constant decrease of barometric pressure and although the pressure exerted by the air is very low in relation to other conditions, various kilometers of elevation above sea level might reduce the resistance within the thoracic cage of those breathing in such conditions [31]. This may be associated with greater lung expandability and therefore greater volume, but it may also be associated with a higher risk of developing pulmonary edema [32].

This such a exposure might triggered several mechanisms that have been associated with greater lung capacities. For instance, having greater FVC is probably linked to anatomical changes that have evolve from centuries of adaptation [6, 33–35]. Some of these evolutionary anatomical adaptations (wider and deeper chest) confer these populations with larger lungs, which result in a greater capacity to accommodate more air. Wider chest and improved pulmonary performance go along with stronger expiration rates. The FEV_1_, was also higher in those living at high altitudes, nevertheless, greater FVC and FEV_1_ was also related with slightly lower FEV_1_/FVC ratio.

These findings have even correlated with similar findings reported in people who rapidly ascend to significant elevations. Sharma et al., in 2007 found that at 3450 m FVC had an initial increase of 9% within the first 24 h followed by a significant decrease in the FVC as well as in FEV_1_ and within the maximal voluntary ventilation. At 5350 m there was a 21% increase in FVC within the first 48 h, with a subsequent decrease as with the other measured values [36]. In relation to age, it appears that the findings on lung capacity are maintained throughout life. Cid-Juarez et al. in 2019 found that inspiratory capacity and forced vital capacity among healthy individuals between 9 and 81 years of age residing above 2240 m elevation presented an enviable increase in these parameters, with the highest peak from 9 to 20 years of age [37]. Another interesting study this time investigating the effects of acute hypoxia on respiratory parameters among young subjects demonstrated that [38]. The "Young Everest study" concluded that at 3500 m above sea level, lung function remained within 7% of baseline among children. They also observed that rapid exposure to high altitude was associated with a significant reduction (up to 23%) in the overall FVC and 16% in FEV_1_ in children [38].

Comparing the spirometric values against predictive values is a fairer comparison. The results of these analysis is to observe how their lungs are performing against of what we would predicted for the same sex, age, weight and height [39, 40]. In Ecuador we do not have defined equations to obtain predetermined pulmonary function values. According to the Third National Health and Nutrition Examination Survey of the American Union (NHANES III), in the case where data is not locally available, the equations to be used are those coming from their closest peers [41]. In Latin America, spirometric studies are relatively scarce. One of the most important projects related to the subject is the Latin American Project for Research in Pulmonary Obstruction (PLATINO). This group found that the predicted values at the pulmonary function level in the Latin American population are like the ones reported for American population that have come originally from Mexico [42]. A study has recently been published in which predictive equations have been performed for each spirometric variable among children living at moderate and high altitude in Colombia, which could be useful for further analysis among children [43].

In this sense, scarce literature is available within the Andean region, nevertheless, Lopez et al., have published an interesting study about the references values among highlanders from South America [18]. They observed higher predictive values in populations living at high altitudes when compared to those predicted values from people located at sea level [18]. Similar results were published by Firoi et al., conducted a study in Central Asia where he compared the Kirghiz population with their medium and low altitude counterparts and observed that FEV_1_ and FVC were lower in the high altitude populations [44].

The objectives of our study were not to identify etiologic factors of chronic obstructive pulmonary disease (COPD), nonetheless with the few patients who had a previous pathologic history we were able to find that both populations had a restrictive pattern in 12.9% with a predominance in the low altitude group. In other high altitude regions, COPD seems to be a significant health problem. In Yanfei Guo et al., publication, COPD in residents living at 2100–4700 m above sea level, at least 8.2% reported pulmonary patterns compatible with COPD and concluded that the prevalence of COPD was inversely related to altitude, a similar conclusion that the one reported by Laniado et al., in 2012 [45, 46].

In relation to the presence of smoking or any history of pulmonary disease, we found that women and men at high altitude smoke more than those living at lower altitude (Table 1). One study tried to address the implications of smoking in subjects who reside but were not necessarily born at high altitude. This 5-year prospective cohort study sought to monitor lung function among individuals exposed to chronic intermittent hypoxia (CIH) working in high altitude mines [47]. They found an annual small but significant decrease in FVC and FEV_1_ among those intermittently exposed to hypobaric hypoxia. They reported that the group of smokers have an earlier deterioration of the pulmonary function than then non-smokers [47].

Our results are the first one in using two genotype-controlled natives living at low and high altitude locations. To our knowledge, this is the first study of this kind and the results of this study can be primarily used for further explore the relationship between chronic hypoxia exposure and pulmonary function among adapted and non-adapted subjects living in the Andes.

## Conclusion

Residents of Oyacachi had greater FVC and FEV_1_ than their peers from the Limoncocha, indicating a greater pulmonary capacity, physiologically plausible according to published literature. Lung size and greater ventilatory capacities could be an adaptive mechanism developed by the highlander in response to hypoxia. Our results support the fact that this difference in FVC and FEV_1_ is a compensatory mechanism towards lower barometric and alveolar partial pressure of oxygen pressure. Although our study is the first to be conducted in two genetically-controlled populations residing at different altitudes, further studies and bigger sampling are needed to understand all the physiological mechanisms behind our results.

## Limitations

The main limitation is that, despite obtaining a significant sample size to carry out this study, not all the population belonging to these indigenous communities that met the inclusion criteria were willing to participate. So, even if it is a small probability, it cannot rule out that the inclusion of the data corresponding to those people who did not participate could produce variations in our results or even alters our interpretation. Another potential weakness is the gender asymmetry in the sample because men were a lower number of participants than women and weight varied significantly among subjects. Finally, limitation is the fact that we do not have local spirometry equations for the correct analysis of the predictive values among highlanders in Ecuador.

## Data Availability

The datasets generated and analysed during the current study are available in the following link https://github.com/covid19ec/Spirometry.
