# Correction: A comparative analysis of lung function and spirometry parameters in genotype-controlled natives living at low and high altitude

**DOI:** 10.1186/s12890-024-03282-5

**Published:** 2024-10-16

**Authors:** Esteban Ortiz-Prado, Sebastián Encalada, Johanna Mosquera, Katherine Simbaña-Rivera, Lenin Gomez-Barreno, Diego Duta, Israel Ochoa, Juan S. Izquierdo-Condoy, Eduardo Vasconez, German Burgos, Manuel Calvopiña, Ginés Viscor

**Affiliations:** 1 One Health Research Group, Faculty of Medicine, Universidad de las Américas, Calle de los Colimes y Avenida De los Granados, 170137 Quito, Ecuador; 2 Department of Cell Biology, Physiology and Immunology, Universidad de Barcelona, Barcelona, Spain; 3 Limoncocha Community Health Unit, Limoncocha, Ecuador; 4 Oyacachi Community Health Unit, Oyacachi, Ecuador; 5 Faculty of Medicine, Universidad de las Américas, Quito, Ecuador

**Correction: BMC Pulmonary Medicine (2022) 22:100**

10.1186/s12890-022-01889-0

Following publication of the original article, it came to the attention of the authors that there were errors in some units in Table 2, Table 3, and Figure 2 (some units were missing whiles others were incorrect). The tables and figure have since been corrected in the published article and the journal would like to refer readers to these new, correct versions of the tables and figure. The authors thank you for reading this erratum and apologize for any inconvenience caused.

